# Improved GAL4 and Tet OFF drivers for *C. elegans* bipartite expression

**DOI:** 10.17912/micropub.biology.000438

**Published:** 2021-09-14

**Authors:** Michael Nonet

**Affiliations:** 1 Washington University School of Medicine, St Louis, MO, USA

## Abstract

The first generation of *C. elegans* GAL4 drivers for bipartite expression function less well than *C. elegans* tet ON/OFF, QF and LexA drivers. The main difference between the GAL4 drivers and the others is the absence of a flexible linker between the DNA binding and activation domain in the GAL4 construct. Addition of a linker to a GAL4-QF construct increased driver potency, while adding linkers to a GAL4-VP64 driver was much less effective. Extending the linker region of the tetR-L-QF driver also increased activity of that driver. The new GAL4 driver makes GAL4/UAS bipartite system activity comparable to the other worm bipartite expression systems.

**Figure 1. GAL4SK and TetR bipartite driver activity improves with a flexible linker:**
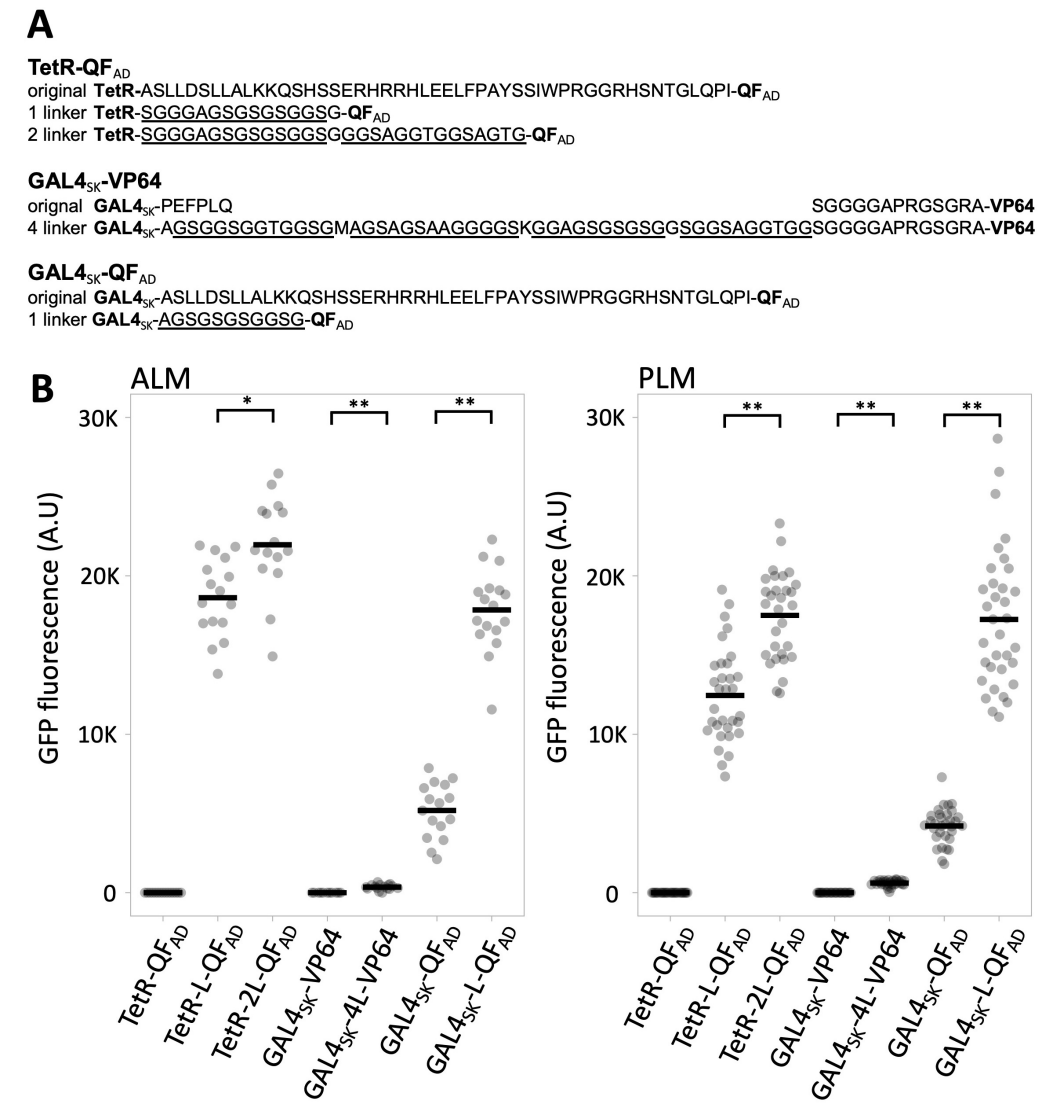
Quantification of GFP expression of various GAL4**_SK_** and TetR bipartite drivers with distinct linkers separating the DNA and activation domains. The *mec-4* promoter was used to express the drivers. An *11X UAS ∆pes-10p GFP-C1* reporter was used to assay GAL4**_SK_** activity and a *7X tetO* ∆pes-10 *GFP-C1* reporter was used to assay TetR activity. See strain list for the exact genotype of animals analyzed. Individual measurements (filled grey circles) and the mean (black bar) are shown. The units are defined identically for ALM and PLM measurements. All of the transgenes also express in AVM and PVM. None of the transgenes express in any other cell types to detectable levels. Strains used: NM5225, NM5467, NM5468, NM5301, NM5470, NM5233 and NM5362. n=15-17 for ALM and 30-34 for PLM. T-test: p*<0.01, ** p<0.0001.

## Description

Several bipartite systems have been described for use with *C. elegans* (Wei *et al.*, 2012; Wang *et al.*, 2017; Wang *et al.*, 2018; Mao *et al.*, 2019; Nonet, 2020)*.* However, little manipulation of the drivers or reporters has been performed to optimize them for *C. elegans*. Mao. et. al. 2019 demonstrated that the QF activation domain is a more potent activator than either VP64 or the hybrid VP64-p65-Rta tripartite activatorVPR. Here, I describe modifications of linker region between the activation domains of both GAL4_SK_ and TetR drivers that increase the activity of these reporters. Although these studies are not comprehensive, I opted to describe them herein because the modification of the GAL4_SK_ driver increases the activity of this driver substantially such that it is now on par with the LexA, TetR and QF2 drivers.

I used an efficient RMCE protocol (Nonet, 2020) to create the transgenic animals. Modified versions of a *mec-4* promoter GAL4_SK_-QF_AD_, GAL4_SK_-VP64 and TetR-QF_AD_ constructs were created in an RMCE integration vector using a Golden Gate cloning approach, then integrated on Chr IV using a standard injection protocol. After outcrossing to the appropriate reporter, the expression level of GFP at steady state in PLM and ALM soma of L4 animals was quantified (Figure 1).

Previously, I described drivers consisting of *C. elegans* codon optimized synthetic GAL4_SK_, TetR and LexA DNA binding domains fused to the QF activation domain based on the observations of Mao. *et al.* 2019. Although the GAL4_SK_ construct was modestly active, the LexA and TetR construct were incapable of activating the reporter (supplemental methods of Nonet, 2020). Replacement of a 49 amino acid portion of central domain of QF, which separates the TetR and LexA DNA binding domains and the QF activation domain in the original constructs, with a 12 amino acid flexible linker converted both into much stronger drivers than the GAL4_SK_-QF_AD_ driver (Nonet, 2020). Here I show that insertion of a similar linker also greatly increases the potency of the GAL4_SK_-QF_AD_ driver. I also extended the linker of the TetR-L-QF_AD_ and this further improved activity of this driver. In Nonet, 2020, I also tested the functionality of a GAL4_SK_-VP64 construct (Wang *et al.* 2017) in single copy and found it was incapable of expressing the GFP reporter. To test if the failure of GAL4_SK_-VP64 was also the result of insufficient domain separation, I inserted a 40 amino acid flexible linker in between GAL4 and VP64. Although the linker containing driver activates transcription, it does so extremely poorly in comparison to the GAL4_SK_-QF_AD_ drivers. I speculate this is likely due to the loss of the MED25 subunit of the mediator complex in the nematode lineage (Grants *et al.*, 2015). VP64 (a 4X VP16) is known to activate transcription in part through interaction with MED25 (Mittler *et al.*, 2003) and the loss of this interaction could account for the observed weak activation properties of VP64 and related activators in worms (Mao *et al.* 2019 and herein).

In addition to the modification of the linker domain of the transgenes I characterize herein, some of the transgenes also differ in other ways that could theoretically impact my conclusions. First, some driver transgenes contain a *tbb-2* 3′ UTR and others use an *act-4* 3′ UTR. I consider it very unlikely these differences impact GFP expression for two reasons. First, my lab has previously demonstrated that *mec-4*promoter driven GFP-C1 transgenes employing the *tbb-2* 3′ UTR and the *act-4* 3′ UTR express at very similar levels in ALM and PLM (Dour and Nonet, 2021). More importantly, I previously demonstrated that the activity of the both a GAL4-QF_AD_ driver and a LexA-L-QF_AD_ driver are insensitive to dosage of the driver (Figure 5 of Nonet, 2020). Specifically, GFP levels observed in GAL4_SK_/+; UAS::GFP ~= GAL_SK_; UAS::GFP. Thus, the level of expression of the driver is unlikely to be determining the GFP signal level. Rather, I speculate that GAL4_SK_ is saturating the UAS binding sites in all transgenes and that the inherent activation properties of the driver determines the expression level.

Second, all drivers were integrated at *jsTi1493* except for the inactive linkerless TetR-QF_AD_ driver. I present data on integration of NMp3730 *(mec-4p tetR-QF*_AD_ *tbb-2 3′ UTR)* at *jsTi1490* (Nonet, 2020). I also previously created insertions of the same plasmid at *jsTi1493* IV and *jsTi1492* II (*jsSi1531* *[mec-4p tetR-QF*_AD_ *tbb-2 3′] IV* and *jsSi1534 [mec-4p tetR-QF*_AD_ *tbb-2 3′] II*; Table S1 and Table S5 in Nonet, 2020). The GFP expression was undetectable in *jsSi1531/+; jsSi1519 [7X tetO ∆pes-10p GFP]/+* and *jsSi1534/+;jsSi1519/+* double heterozygote animals. However, only the *jsTi1490* transgene was homozygosed with the reporter. Thus, I quantified that insertion. However, this difference is highly unlikely to impact my findings as the other two insertions are qualitatively completely inactive.

The improvements to GAL4_SK_-QF_AD_ by insertion of a flexible linker is an important addition to the *C. elegans* bipartite expression toolkit since the GAL4 system is so extensively developed in *Drosophila*. Using multi-copy lines and a VP64 activator, Wang *et al.* (2018) have already shown that the split GAL4_SK_ system is functional in worms. Incorporation of a similar flexible linker and a QF activation domain into those tools should permit development of a robust single copy split-GAL4_SK_ system. Other GAL4 system tools previously developed for *Drosophila* such as GAL80ts and GAL4-PR tools (Caygill and Brand, 2016) which provide temporal control in addition to spatial control could also easily be incorporated into the worm toolbox.

In addition, further manipulation of the size and properties of the linker domain separating the DNA binding domain and activation domains could yield drivers with even stronger activation proper which would likely also be applicable to the LexA, QF and Tet ON/OFF driver/reporter systems.

## Methods

**Methods**

*C. elegans* was maintained on NGM agar plates spotted with OP50 at 22.5°C or at 25°C during the RMCE protocol.

**RMCE transgenesis**

Inserts were cloned into pLF3FShC (Addgene # 153083; Nonet, 2020) and injected at ~50 ng/µl into *jsTi1493* young adults. Integrants were identified and isolated as described in detail in Nonet (2020). The GAL4_SK_ drivers were crossed to *jsSi1518 [11X UAS ∆pes-10 GFP-C1]* and the TetR strains were crossed to *jsSi1543 [7X tetO ∆mec-7p GFP-C1]*.

**Microscopy**

For quantification of GFP signals, homozygous L4 hermaphrodite animals were mounted on 2% agar pads in a 2 µl drop of 1mM levamisole in phosphate buffered saline and imaged on an Olympus (Center Valley, PA) BX-60 microscope equipped with a Qimaging (Surrey, BC Canada) Retiga EXi monochrome CCD camera, a Lumencor AURA LED light source, Semrock (Rochester, NY) GFP-3035B and mCherry-A-000 filter sets, and a Tofra (Palo Alto, CA) focus drive, run using micro-manager 2.0ß software (Edelstein *et al.*, 2014) using a 40X air lens at 20% LED power with 50 ms exposures. PLM soma and ALM soma signals were quantified using the FIJI version of ImageJ software (Schindelin *et al.*, 2012) as described in Nonet (2020).

**Plasmid constructions**

Integration vectors were assembled using Golden Gate (GG) reactions as described in Nonet (2020). Other plasmids were constructed using standard cloning techniques.

The following were used:

**NMp3055 DR274 U6** (Table S3 and Supplemental methods of Nonet, 2020)

**NMp3401 DR274 CT linker**

NMp3055 was digested with EcoRI and HindIII and the double stranded (ds) oligonucleotide NMo5948/49 was ligated into the vector.

**NMp3403 DR274 CT-FP linker**

NMp3055 was digested with EcoRI and HindIII and the ds oligonucleotide NMo5952/53 was ligated into the vector.

**NMp3498 DR274 FP linker**

NMp3403 was modified by DpnI-mediated mutagenesis using oligonucleotides NMo6120/21.

**NMp3610 syn Gal4_SK_ DB** (Table S3 and Supplemental methods of Nonet, 2020)

**NMp3617 pSAP mec-4p GAL_SK_-VP64** (Table S3 and Supplemental methods of Nonet, 2020)

**NMp3643 pLF3FShC** Available at Addgene. (Table S3 and Supplemental methods of Nonet, 2020)

**NMp3735 DR274 5’arm- CT mec-4p** (Table S3 and Supplemental methods of Nonet, 2020)

**NMp3736 DR274 TGG GGT mec-4p** (Table S3 and Supplemental methods of Nonet, 2020)

**NMp3751 DR274 AAG GTA act-4 3’UTR** (Table S3 and Supplemental methods of Nonet, 2020)

**NMp3777 DR274 3’arm tbb-2 UTR** (Table S3 and Supplemental methods of Nonet, 2020)

**NMp3808 DR274 CT-NT linker-QF_AD_** (Table S3 and Supplemental methods of Nonet, 2020)

**NMp3821 DR274 FP tetR(iS)-L-QF_AD_** (called DR274 FP tetR(iS)-L-QF in Table S3 and Supplemental methods of Nonet, 2020)

**NMp3876 pLF3FShC mec-4p GAL4_SK_-L-QF_AD_**

NMp3736, NMp3610, NMp3808 and NMp3777 were co assembled into NMp3643 using a SapI GG reaction.

**NMp4045 DR274 FP tetR-LL-QF_AD_**

NMp3821 was amplified with oligonucleotides NMo6867 and NMo7056, DpnI digested, purified, kinased, and religated.

**NMp4048 pLF3FShC mec-4p tetR-LL-QF_AD_ act-4**

NMp3735, NMp4045, and NMp3751 were co assembled into NMp3643 using a SapI GG reaction.

**NMp4049 pLF3FShC mec-4p GAL4_SK_-L4-VP64 tbb-2 3’UTR**

NMp3736, NMp3610, NMp3401, NMp3498, NMp3777, the ds oligonucleotide NMo6866/67, and VP64 as a PCR product amplified from NMp3617 using oligonucleotides NMo6404/7057 were co assembled into NMp3643 using a SapI GG reaction.

**Oligonucleotides**

**Table:** 

**NMo number**	**Sequence**
5948	AATTGCTCTTCgGCGGGCAGCGGTGGCAGTGGAGGTACCGGCGGAAGCGGTATGcGAAG
5949	AGCTCTTCgCATACCGCTTCCGCCGGTACCTCCACTGCCACCGCTGCCCGCcGAAGAGC
5952	AATTGCTCTTCgGGTGGCAGCGCTGGAGGTACCGGCGGTAGTGCCGGAGGCACGcGAAG
5953	AGCTCTTCgCGTGCCTCCGGCACTACCGCCGGTACCTCCAGCGCTGCCACCcGAAGAGC
6120	GCTCTTCAATGGCCGGCTCCGCCGGCTCT
6121	CGGAGCCGGCCATTGAAGAGCAATTCAAAAATCATACC
6404	CAAGCTCTTCGCGTTTAGTTAATCAGCATGTCCAGG
6866	AAGGGAGGAGCGGGTTCTGGATCTGGATCTGGAGGTTCC
6867	ACCGGAACCTCCAGATCCAGATCCAGAACCCGCTCCTCC
7056	GGTGGCAGCGCTGGAGGTACCGGCGGTAGTGCCGGAACGggtcgtcaacttga
7057	AATGCTCTTCaGGTGGCAGCGCTGGAGGTACCGGCGGTTCTGGTGGCGGAGGG

**Transgenes**

**Table:** 

**Name**	**Description**	**Full Designation**	**Comments**
*jsTi1493 IV*	*Chr IV landing site*	*jsTi1493 [mosL loxP mex-5p FLP sl2 mNeonGreen rpl-28p FRT GFP-HIS-58 FRT3 mosR] IV*	Nonet, 2020
*jsSi1515 IV*	*mec-4p::GAL4_SK_-QF_AD_*	*jsTi1493 jsSi1515 [mosL loxP mec-4p GAL4_SK_-QF_AD_ tbb-2 3′ FRT3 mosR] IV*	Nonet, 2020
*jsSi1516 IV*	*mec-4p::tetR-QF_AD_*	*jsTi1490 jsSi1516 [mosL loxP mec-4p tetR-QF_AD_ tbb-2 3′ FRT3 mosR] IV*	Nonet, 2020
*jsSi1518 I*	*11X UAS::GFP*	*jsTi1453 jsSi1518 [mosL loxP 11X UAS ∆pes-10p GFP-C1 tbb-2 3′ FRT3 mosR] I*	Nonet, 2020
*jsSi1519 I*	*7X tetO ∆pes-10::GFP*	*jsTi1453 jsSi1519 [mosL loxP 7X tetO ∆pes-10p GFP-C1 tbb-2 3′ FRT3 mosR] I*	Nonet, 2020
*jsSi1525 IV*	*mec-4p::GAL4_SK_-VP64*	*jsTi1493 jsSi1525 [mosL loxP mec-4p GAL4_SK_-VP64 tbb-2 ‘3 FRT3 mosR] IV*	Nonet, 2020
*jsSi1543 I*	*7X tetO ∆mec-7p::GFP*	*jsTi1453 jsSi1543 [mosL loxP tetO 7X ∆mec-7p GFP-C1 tbb-2 3′ FRT3 mosR] I*	Nonet, 2020
*jsSi1560 IV*	*mec-4p::tetR-L-QF_AD_*	*jsTi1493 jsSi1560 [mosL loxP mec-4p tetR-L-QF_AD_ act-4 3′ FRT3 mosR] IV*	Nonet, 2020
*jsSi1588 IV*	*mec-4p::GAL4_SK_-L-QF_AD_*	*jsTi1493 jsSi1588 [mosL loxP mec-4p GAL4_SK_-L-QF_AD_ act-4 3′ FRT3 mosR] IV*	RMCE insertion of NMp3876 into *jsTi1493*
*jsSi1661 IV*	*mec-4p::tetR-L-QF_AD_*	*jsTi1493 jsSi1661 [mosL loxP mec-4p tetR-LL-QF_AD_ act-4 3′ FRT3 mosR] IV*	RMCE insertion of NMp4048 into *jsTi1493*
*jsSi1664 IV*	*mec-4p::GAL4_SK_-L4-VP64*	*jsTi1493 jsSi1664 [mosL loxP mec-4p GAL4_SK_-L4-VP64 tbb-2 3’ FRT3 mosR] IV*	RMCE insertion of NMp4049 into *jsTi1493*

**Worm Strains**

**Table:** 

**NM strain**	**Genotype**	**Source**
5179	*jsTi1493 [mosL loxP mex-5p FLP sl2 mNeonGreen rpl-28p FRT GFP-HIS-58 FRT3 mosR] IV*	Nonet, 2020; CGC
5213	*jsTi1453 jsSi1518 [mosL loxP 11X UAS ∆pes-10p GFP-C1 tbb-2 3′ FRT3 mosR] I; him-8(e1489) IV*	Nonet, 2020
5214	*jsTi1453 jsSi1519 [mosL loxP 7X tetO ∆pes-10p GFP-C1 tbb-2 3′ FRT3 mosR] I; him-8(e1489) IV*	Nonet, 2020
5225	*jsTi1453 jsSi1519 [mosL loxP tetO 7X ∆pes-10p GFP-C1 tbb-2 3′ FRT3 mosR] I; jsTi1490 jsSi1516 [mosL loxP mec-4p tetR-QF_AD_ tbb-2 3′ FRT3 mosR] IV*	This work
5233	*jsTi1453 jsSi1518 loxP UAS 11X ∆pes-10p GFP-C1 tbb-2 3′ FRT3 mosR] I; jsTi1493 jsSi1515 [mosL loxP mec-4p GAL4_SK_-QF_AD_ tbb-2 3’ FRT3 mosR] IV*	Nonet, 2020; CGC
5264	*jsTi1453 jsSi1543 [mosL loxP tetO 7X ∆mec-7p GFP-C1 tbb-2 3′ FRT3 mosR] I; him-8(e1489) IV*	Nonet, 2020
5295	*jsTi1493 jsSi1560 [mosL loxP mec-4p tetR-L-QF_AD_ act-4 ‘3 FRT3 mosR] IV*	Nonet, 2020
5301	*jsTi1453 jsSi1518 [mosL loxP UAS 11X ∆pes-10p GFP-C1 tbb-2 3′ FRT3 mosR] I; jsTi1493 jsSi1525 [mosL loxP mec-4p GAL4_SK_-VP64 tbb-2 3’ FRT3 mosR] IV*	Nonet, 2020
5353	*jsTi1493 jsSi1588 [mosL loxP mec-4p GAL4_SK_-L-QF_AD_ act-4 3′ FRT3 MosR] IV*	This work
5362	*jsTi1453 jsSi1518 [mosL loxP UAS 11X ∆pes-10 GFP-C1 tbb-2 3′ FRT3 mosR] I; jsTi1493 jsSi1588 [mosL loxP mec-4p GAL4_SK_-L-QF_AD_ act-4 3′ FRT3 mosR] IV*	This work
5467	*jsTi1453 jsSi1543 [mosL loxP tetO 7X ∆mec-7p GFP-C1 tbb-2 3′ FRT3 mosR] I; jsTi1493 jsSi1560 [mosL loxP mec-4p tetR-L-QF_AD_ act-4 3’ FRT3 mosR] IV*	This work
5468	*jsTi1453 jsSi1543 [mosL loxP tetO 7X ∆mec-7p GFP-C1 tbb-2 3′ FRT3 mosR] I; jsTi1493 jsSi1661 [mosL loxP mec-4p tetR-LL-QF_AD_ act-4 3′ FRT3 mosR 3′ ] IV*	This work
5470	*jsTi1453 jsSi1518 [mosL loxP UAS 11X ∆pes-10 GFP-C1 tbb-2 3′ FRT3 mosR] I; jsTi1493 jsSi1664 [mosL loxP mec-4p GAL4_SK_-L4–QF_AD_ tbb-2 3’ FRT3 mosR] IV*	This work

## Reagents

Plasmids and worm strains are available by request from MLN and will be submitted to Addgene and the *Caenorhabditis* Genetics Center if demand levels warrant it.

## References

[R1] Caygill EE, Brand AH (2016). The GAL4 System: A Versatile System for the Manipulation and Analysis of Gene Expression.. Methods Mol Biol.

[R2] Dour S, Nonet M (2021). Optimizing expression of a single copy transgene in *C. elegans*.. MicroPubl Biol.

[R3] Edelstein AD, Tsuchida MA, Amodaj N, Pinkard H, Vale RD, Stuurman N (2014). Advanced methods of microscope control using μManager software.. J Biol Methods.

[R4] Grants JM, Goh GY, Taubert S (2015). The Mediator complex of Caenorhabditis elegans: insights into the developmental and physiological roles of a conserved transcriptional coregulator.. Nucleic Acids Res.

[R5] Mao S, Qi Y, Zhu H, Huang X, Zou Y, Chi T (2018). A Tet/Q Hybrid System for Robust and Versatile Control of Transgene Expression in C. elegans.. iScience.

[R6] Mittler G, Stühler T, Santolin L, Uhlmann T, Kremmer E, Lottspeich F, Berti L, Meisterernst M (2003). A novel docking site on Mediator is critical for activation by VP16 in mammalian cells.. EMBO J.

[R7] Nonet ML (2020). Efficient Transgenesis in *Caenorhabditis elegans* Using Flp Recombinase-Mediated Cassette Exchange.. Genetics.

[R8] Schindelin J, Arganda-Carreras I, Frise E, Kaynig V, Longair M, Pietzsch T, Preibisch S, Rueden C, Saalfeld S, Schmid B, Tinevez JY, White DJ, Hartenstein V, Eliceiri K, Tomancak P, Cardona A (2012). Fiji: an open-source platform for biological-image analysis.. Nat Methods.

[R9] Wang H, Liu J, Gharib S, Chai CM, Schwarz EM, Pokala N, Sternberg PW (2016). cGAL, a temperature-robust GAL4-UAS system for Caenorhabditis elegans.. Nat Methods.

[R10] Wang H, Liu J, Yuet KP, Hill AJ, Sternberg PW (2018). Split cGAL, an intersectional strategy using a split intein for refined spatiotemporal transgene control in *Caenorhabditis elegans*.. Proc Natl Acad Sci U S A.

[R11] Wei X, Potter CJ, Luo L, Shen K (2012). Controlling gene expression with the Q repressible binary expression system in Caenorhabditis elegans.. Nat Methods.

